# Direct and Indirect Effects of Herbicides on Insect Herbivores in Rice, *Oryza sativa*

**DOI:** 10.1038/s41598-019-43361-w

**Published:** 2019-05-06

**Authors:** Emily C. Kraus, Michael J. Stout

**Affiliations:** Louisiana State University AgCenter, Baton Rouge, USA

**Keywords:** Abiotic, Agroecology, Plant physiology

## Abstract

Densities of insect pests in agricultural communities may be affected by herbicides commonly used for weed management via several routes. First, herbicides may cause direct mortality to insects present both during and immediately following application. Second, herbicides may induce plant defenses that increase resistance to insect herbivores. Third, herbicides may alter the quantity and composition of weed populations, which in turn may change the structure of insect communities found subsequently in the crop. This study was designed to investigate the effects of an array of herbicides on the densities of several major pests found in rice in the southern United States. These pests included the rice water weevil, *Lissorhoptrus oryzophilus* Kuschel (Coleoptera: Curculionidae), the rice stinkbug, *Oebalus pugnax* (Fabricius) (Hemiptera: Pentatomidae), and a stemborer complex comprised of three lepidopteran species (Lepidoptera: Crambidae). Insects directly exposed to herbicides experienced high mortality; while those fed leaf material that had been exposed to herbicides did not. Herbicide application did not significantly increase resistance in rice to subsequent herbivore infestation. Results provided modest support for the third hypothesis represented by positive correlations between weed densities and insect pest densities.

## Introduction

Weeds and insect pests are both critical constraints on rice yields globally^1^. In the southern U.S. where rice is an important crop as many as 80 weed species are present in commercial rice fields. These weeds include red rice and barnyard grass, which can reduce yields by as much as 80%^2^. Chemical herbicides are the most common tactic for controlling weeds in agriculture^3^. Herbicides can be applied from before emergence to mid-season to manage weed pests, but the majority of herbicides in southern U.S. rice are applied early season, prior to permanent flood^2^. Red rice has long been the primary driver of weed management practices, and historically was controlled mainly by water management. This changed with the introduction in 2002 of technology for controlling red rice using rice varieties tolerant of herbicides containing imidazolinones such as ‘Newpath’. In addition a variety of herbicides are available to growers depending on the target weed species and timing the herbicides are needed^2^.

Herbicide applications made to agricultural fields to control weeds may also affect insect pests. There are at least three routes by which herbicides may impact insect communities. First, herbicide applications may cause direct mortality of insects present in fields at the time of their application^4–8^. Second, herbicide applications can induce resistance in crop plants, thereby affecting insect communities indirectly^9–11^. Herbicide-induced plant resistance is possible because herbicides can act as stressors to crop plants, and may increase expression of defense-related genes, which can in turn alter densities of pest populations on the crop. Finally, herbicides by design alter the composition of plant communities in agricultural fields, and because weeds can serve as alternate hosts for many crop pests, removal of weeds by herbicides may have an effect on the density and composition of insect communities^12^. Examples of all three routes of herbicide effect are found in the scientific literature^13^. Norris and Kogan (2000) reviewed the various interactions among weeds, arthropod pests, and their natural enemies, and provided dozens of examples of resource and habitat-driven interactions between weeds and insects, including effects of weed management practices such as tillage and herbicide use. Their work suggests that interactions between weeds and arthropods can be direct or indirect, can involve trophic cascades, and can be affected by agricultural management practices. Management practices can affect interactions between weeds and arthropods by directly affecting a pest, or by altering aspects of crop growth^13^.

There are numerous examples in the literature of direct mortality of pest herbivores from herbicide applications. The herbicide AAnetos L caused significant mortality to Collembola at low doses. Both the active ingredient (2,4,5-T isooctylester) and the inert ingredients of the herbicide were found to be toxic to the hexapods via direct contact^4^. The herbicide glufosinate-ammonium was highly toxic to nymphs and adults of the mite species *Amblyseius womersleyi* Schicha (Acari: Phytoseiidae), *Phytoseiulus persimilis* Athias-Henriot (Acari: Phytoseiidae), and *Tetranychus urticae* Koch (Acari: Tetranychidae)^5^. Imazethapyr and quizalofop ethyl, the active ingredients in the herbicides Pursuit 10EC and Tergasuper 5EC, significantly reduced survival of *Spilarctia obliqua* (Walker) (Lepidoptera: Erebidae) larvae^6^. The same study showed that 2, 4-dichlorophenoxyacetic acid (2,4-D) was non-toxic to the insect, a finding further supported by Haag^7^. In this latter study, exposure to 2,4-D, diquat, and glyphosate had no effect on survival of the water hyacinth weevils *Neochetina eichhorniae* Warner (Coleoptera: Curculionidae) and *N*. *bruchi* Hustache (Coleoptera: Curculionidae)^7^.

A more recent study showed that herbicides with 2,4-D and diquat as active ingredients negatively affected survival of both *N*. *eichhorniae* (Warner) and *Eccritotarsus catarinensis* (Carvalho) (Hemiptera: Miridae). Mortality was as high as 80% for the latter species, higher than mortality caused by herbicides containing glyphosate for both insects^8^. This study also noted that surfactants included in formulations increased insect mortality. The diversity of results in the literature suggests that the effects of herbicides on insect mortality are dependent upon active ingredient, formulation, and the insect species^4–8^.

Herbivore-induced resistance in plants has also been noted in numerous studies^14–17^. Rice was the focus of some of these studies and is known to be inducible by herbivore feeding and by application of hormones^14–17^. This wealth of prior research with rice suggests that this crop is an appropriate system with which to explore the importance of indirect effects of herbicides on pest populations. Herbicide-induced resistance in rice has been shown in a report involving 2,4-D which induced resistance in rice to the striped stem borer, *Chilo suppressalis* (Walker) (Lepidoptera: Crambidae)^9^. This same study also provided evidence that 2,4-D application resulted in an increase in volatile production and an increase in activities of trypsin proteinase inhibitor and that these changes were mediated by jasmonic acid and ethylene. Finally, plants treated with 2,4-D in this study were more attractive to *Anagrus nilaparvatae* (Pang et Wang) (Hymenoptera: Mymaridae), a parasitoid, and supported lower growth rates of *C*. *suppressalis* (Walker)^9^.

In a second study in rice, application of the herbicide diclofop-methyl induced systemic acquired resistance (SAR) via the salicylic acid (SA) pathway and not the jasmonic acid (JA) pathway^18^. The SA pathway is more strongly associated with pathogen resistance while the JA pathway is more strongly associated with resistance to chewing herbivores^19^. Therefore, the signaling pathways induced and the pests affected by herbicide applications likely depend on the herbicide used.

In another study, in soybean, *Glycine max* (L.), and nodding plume thistle, *Carduus nutans* (L.), effects of dicamba on two insect species were investigated. The herbicide was non-toxic when directly applied to *Helicoverpa zea* (Boddie) (Lepidoptera: Noctuidae) or when insects were fed on dicamba-treated soybean. There were significant negative indirect effects on *Vanessa cardui* (Linnaeus) (Lepidoptera: Nymphalidae) larvae that had fed on thistle treated with the herbicide. Larvae fed dicamba-treated leaves had significantly lower larval and pupal masses compared to controls^10^. Herbicide application also resulted in reduced plant growth, which may have resulted in limitations in food availability for larvae. An additional example of herbicide-induced resistance involves the herbicide S‐ethyldipropylthiocarbamate (EPTC), which has been shown to induce resistance in cabbage (*Brassica oleracea* Linnaeus) to three insect pests including diamondback moth, *Plutella xylostella* (Linnaeus) (Lepidoptera: Plutellidae), imported cabbage worm, *Pieris rapae* (Linnaeus) (Lepidoptera: Pieridae), and cabbage looper, *Trichoplusia ni* (Hubner) (Lepidoptera: Noctuidae). The herbicide induced a glossy phenotype that reduced feeding by first instars of all three species^11^.

Finally, insect communities may be indirectly affected by herbicide use via eliminating alternate hosts for insect pests, resulting in decreased insect pest densities. Conversely, poor weed management can lead to a lack of insect control by providing additional food sources for pests^12^. Herbicide drift, from 2,4-D and dicamba, has been noted to affect plant and arthropod communities. Field-based experiments carried out over multiple years showed that dicamba caused a decline in forb cover and consequently several pest herbivores. Increases in density of one root feeding species and one beneficial species were also observed^12^.

Rice agroecosystems in the southern U.S. experience infestations of insect pests at all crop growth stages, and all three routes of herbicide effects discussed above could potentially affect these herbivores. The major pests in most rice fields in the southern U.S. are the rice water weevil (RWW), *Lissorhoptrus oryzophilus* Kuschel, a complex of stem-boring lepidopterans, and the rice stinkbug. Each of these pests specializes on grasses. The RWW is an early and mid-season coleopteran pest of rice. Adults are present in rice throughout the season but are more numerous in early season rice^20^. Adults feed on leaves of rice, but this form of feeding causes relatively little economic damage. Flooding triggers oviposition by females, and for that reason larvae are only present in high numbers after flooding^21^. Larval feeding reduces plant tillering and shoot growth, as well as root biomass. Root pruning by larvae reduces panicle densities and grain harvest weights^22^. Due to timing of the occurrence of RWW in rice fields, this insect pest could be affected directly via herbicide toxicity or by herbicide-induced effects on plant resistance, because it is present in fields when the majority of herbicides are applied.

Mid- to late-season rice is also host to several stem boring species. The insects in this complex include the sugarcane borer, *Diatraea saccharalis* (Fabricus), the rice stalk borer, *Chilo plejadellus* Zinken, and the Mexican rice borer, *Eoreuma loftini* (Dyar). These insects primarily infest mid-tillering and reproductive stages of rice. Larval feeding can be economically damaging in multiple rice stages. This damage occurs as the larvae bore into and tunnel through the rice stems. In the reproductive stage larval injury may cause whiteheads, which result in unfilled grains and overall yield loss^23^. Stemborers might be affected directly by herbicides, but due to the timing of their occurrence in fields they are more likely to be affected by herbicide-induced effects or by alterations in weed communities^24^.

*Oebalus pugnax* (Fabricus), the rice stinkbug, is native to North America, where it is a pest of wheat, sorghum, and rice^25^. This insect enters rice fields late in the season after rice has begun heading. Adults and late instar nymphs cause damage to rice by removing the contents of developing grains with their piercing-sucking mouthparts, thereby negatively affecting rice yields and the quality of grains^26–28^. Because these insects do not enter fields until at least 60 days after most herbicides are applied, direct mortality from herbicides is extremely unlikely. Although herbicides have been shown to alter susceptibility of rice to piercing-sucking insects in past studies (such as the brown planthopper^29,30^), the temporal separation between herbicide applications and stink bug infestation makes it most likely that rice stink bugs would be affected by changes in the weed community resulting from herbicide applications.

This study had three objectives. The first objective was to determine if any of seven herbicides commonly used in southern U.S. rice affected RWW mortality. Potential direct effects of herbicides on mortality of stemborers and rice stink bugs were not tested because the pattern of occurrence of these pests in rice fields strongly reduces the probability of direct exposure to herbicide applications. The second objective was to determine if these herbicides induced resistance in the rice plant thereby reducing subsequent pest densities of RWW, rice boring lepidopterans, or rice stinkbugs, and altering subsequent yield. Evidence from most prior studies suggests induction of the jasmonic acid pathway by herbicides in rice^9^. This pathway is associated with resistance to chewing herbivores, making it more likely that herbicide-induced resistance would affect RWW and stemborers. The third objective was to determine if reductions in density of weeds and alterations in weed communities due to herbicide application altered densities of insect pests.

## Results

### RWW mortality experiments (objective 1)

**Figure 1:**
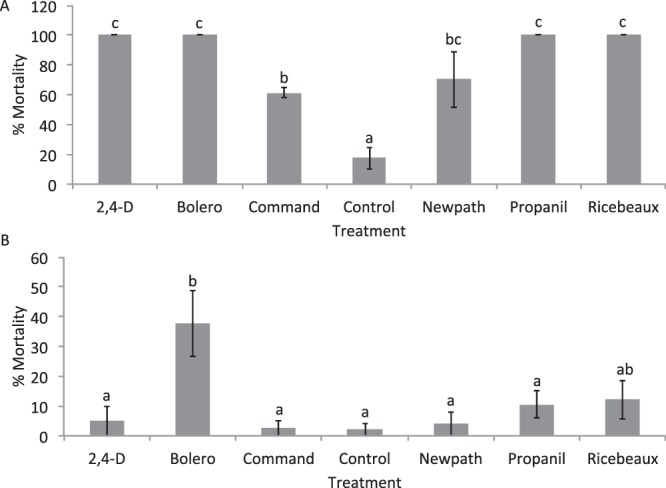
Mean mortalities (% mortality ± SE) of RWW adults by (**A**) direct topical application of herbicides to RWW adults and (**B**) contact with/consumption of herbicide-treated leaf material. Bars accompanied by different letters represent means that differed significantly by Tukey’s HSD test at α < 0.05.

Direct topical application of herbicides to RWW adults caused substantial mortality of insects (F = 16.68; df = 6,21; P < 0.0001) (Fig. 1A). Mortality was higher than in controls for all herbicide treatments. The herbicides 2,4-D, Bolero, Propanil, and Ricebeaux caused 100% mortality. The herbicides Command and Newpath caused 60–70% mortality, significantly higher than the control, but mortality caused by Command was significantly lower than most of the other herbicides.

Results of feeding assays in which RWW adults fed on rice leaves immediately after treating leaves with herbicide showed a significant overall effect of herbicide treatment on RWW mortality (F = 4.67; df = 6,21; P = 0.004), but mortality was only significantly higher than controls for RWW adults that fed on Bolero-treated material (Fig. 1B).

### Field experiments (objectives 2 and 3)

Results from three small-plot field experiments conducted over two growing seasons were used to investigate the relative importance of herbicide-induced resistance (Objective 2) and herbicide-induced changes in weed communities (Objective 3) in influencing populations of insect pests in rice. Multivariate analyses including Canonical Correlation Analysis (CCA) and MANOVA were used to analyze results. Although these analyses could not definitively separate the two routes by which herbicide applications could indirectly affect pest populations, correlations of pest densities with herbicide injury and weed control were used to explore the most likely routes for each pest. In the presentation of results that follows, correlations of pest densities with herbicide injury (Objective 2) are presented before correlations of pest densities and weed control (Objective 3).

In the analysis for each of the three experiments, the first canonical correlation was significant or marginally significant, but subsequent canonical correlations were not significant in any of the three analyses. In the experiment in 2015, the first canonical correlation was 0.839 (F = 2.53; df = 12, 45.27; P = 0.012), with 94.13% of variance explained by the two variates. The canonical cross loading values indicated that in three of four core samplings (RWW1, RWW2, and RWW4) densities of RWW were negatively correlated with injury at seven days post-application (Injury1) (Objective 2) and weed control at both rating dates (Weed1 and Weed2) (Objective 3) (Table 1).

**Table 1:** Canonical cross loading values for one experiment in 2015 and two experiments in 2016 are presented.

Response Variable	Correlation Cross Loading Values for each Experiment		
	2015	2016 (1)	2016 (2)
Injury1	0.637	−0.3453	0.547
Injury2	N/A	−0.2532	0.466
Weed1	0.702	−0.1256	−0.072
Weed2	0.726	0.4455	−0.307
RWW1	−0.572	0.0315	0.306
RWW2	−0.559	0.3205	0.024
RWW3	0.187	N/A	N/A
RWW4	−0.369	N/A	N/A
Whitehead	N/A	−0.2621	0.288
Stinkbug	N/A	−0.1466	0.171
Yield	N/A	0.395	−0.552

In the first experiment of 2016, the first canonical correlation was 0.630 (F = 2.07; df = 20,100.45; P = 0.001), with 48.2% of variance explained by the two variates. Consistent with results from 2015 the canonical cross loading values indicated that injury measured at seven and 21 days post-application (Injury1 and Injury 2) (Objective 2) was negatively correlated with RWW densities (RWW1 and RWW2). Canonical correlation values indicated that injury was positively correlated with whitehead (Whitehead) and rice stink bug (Stinkbug) densities. The relationship between RWW densities and densities of weeds was more complex (Objective 3). RWW densities were negatively correlated with weed control at seven days post-application, but a positive correlation was found for weed control ratings 21 days post-application. Densities of whiteheads and rice stink bug were positively correlated with weed control at seven days post-application, but were negatively correlated with weed control at 21 days post-application. Finally, canonical correlation values indicated that injury at seven and 21 days post-application (Injury1 and Injury 2) was negatively correlated with yield. Weed control at seven days post-application was negatively associated with yield while the opposite relationship was found using ratings of weed control at 21 days post-application (Table 1).

In the second experiment of 2016, the first canonical correlation was 0.643 and was marginally significant (F = 1.60; df = 20, 133.61; P = 0.061), with 78.8% of variance explained by the two variates. The canonical cross loading values indicated that densities of insects (RWW1, RWW2, Whitehead, and Stinkbug) were positively correlated with injury (Injury1 and Injury2) (Objective 2), but negatively correlated with weed control (Weed1 and Weed2) (Objective 3). Yield was negatively correlated with injury (Injury1 and Injury2), but positively correlated with weed control (Weed1 and Weed2) (Table 1).

Simple linear regressions showed there were no significant relationships between injury and insect densities (Objective 2) in 2015 or the first experiment in 2016, but there were significant relationships in the second experiment performed in 2016. In this latter experiment, RWW densities (RWW1) (F = 4.07; df = 1,47; P = 0.050), and whitehead densities (Whitehead) (F = 3.91; df = 1,47; P = 0.054) were positively correlated with injury (Injury1) to rice.

**Figure 2:**
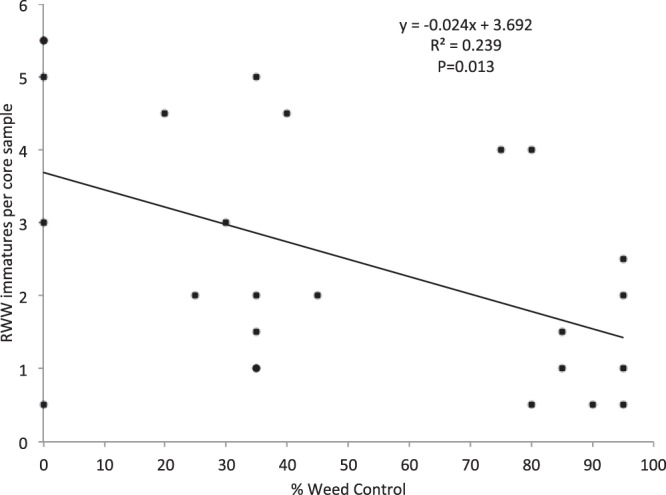
Regression showing the relationship between percent weed control and immature RWW density. The analysis showed a decrease in immature RWW densities as percent weed control increased.

Simple linear regressions showed the relationships between insect densities and weed control (Objective 3) were somewhat consistent among the three experiments. In 2015 and the first experiment of 2016, RWW densities were negatively associated with weed control. In 2015, the relationship between weed control rating at seven days post-application (Weed1) and densities of RWW on the first core sampling date (RWW1) was significant (F = 7.23; df = 1,23; P = 0.013) (Fig. 2). Similarly, densities of RWW immatures in the first core sampling (RWW1) were negatively correlated with weed control at 21 days post-application (Weed2) (RWW1 F = 4.38; df = 1,23; P = 0.048). All other combinations of core samplings and weed control ratings in 2015 showed non-significant relationships.

In the first experiment of 2016, densities of RWW in the second core sampling and percent weed control (Objective 3) at 21 days post-application were significantly positively correlated (F = 12.88; df = 1,44; P = 0.008). Densities of RWW at the first core sampling, rice stink bug densities, and whitehead densities showed no significant relationship to weed control ratings. In the second experiment of 2016 there were no significant regressions among weed control ratings and insect densities.

**Figure 3:**
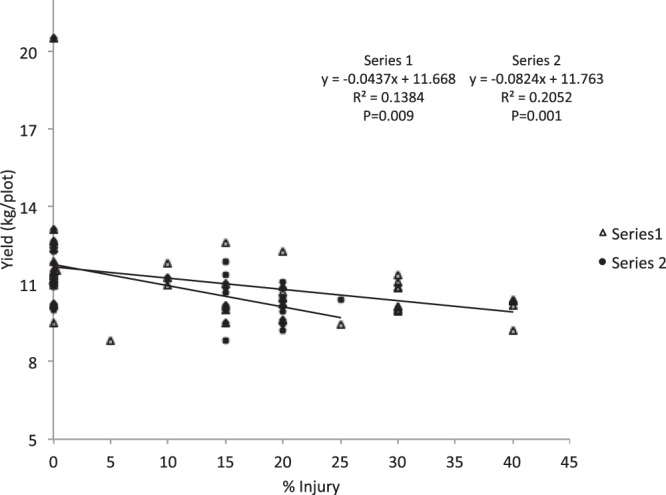
Regression showing the relationship between percent injury to rice (% Injury) and yield (kg/plot). The regression lines show that as percent injury per plot increases the average yield per plot decreases. Series 1 shows the relationship between yield and injury rating at seven days post-application (Injury1). Series 2 shows the relationship between yield and injury rating at 21 days post-application (Injury2).

**Figure 4:**
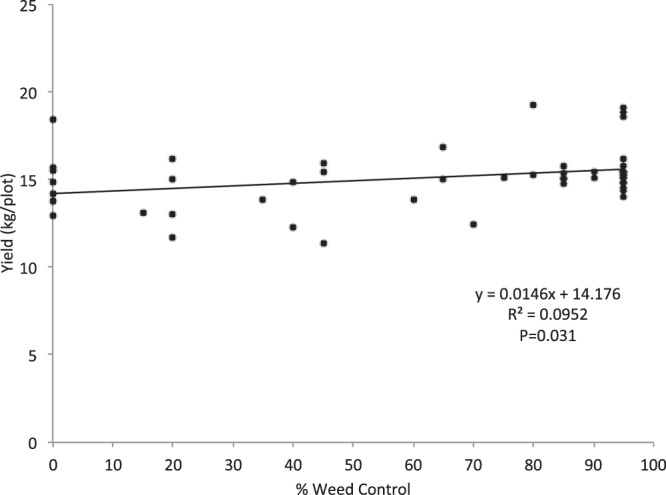
Regression showing the relationship between weed control rating at 21 days post-application and average yield per plot. Yields were found to increase as weed control increased.

Simple linear regression also showed that yields were significantly correlated with injury level and weed control. In the first experiment of 2016, for both injury ratings, there was a negative correlation between injury ratings and yields (Injury1 F = 7.55; df = 1,47; P = 0.009)(Injury2 F = 12.13; df = 1,47; P = 0.001) (Fig. 3). In the second experiment of 2016, results showed that yields were significantly negatively correlated with injury for both the first rating seven days post-application (Injury1) and the second rating 21 days post-application (Injury2) (F = 12.09; df = 1,47; P = 0.001; F = 8.59; df = 1,47; P = 0.005)(data not shown). Finally, yields were positively correlated with percent weed control at 21 days post-application (Weed2) (F = 4.94; df = 1,47; P = 0.031) (Fig. 4), but the relationship was not significant when ratings from 7 days post-application (F = 0.61, df = 1,47; P = 0.440) were used.

For all three field experiments, the MANOVA testing the overall effect of herbicide treatment on response variables was significant; 2015 (F = 10.37; df = 28, 51.9; P < 0.001), 2016(1) (F = 3.47; df = 54, 126.97; P < 0.0001), 2016(2) (F = 4.68; df = 54,177.96; P < 0.0001); with all pairwise contrasts of herbicide treatments to the control significant as well (Table 2) (Objectives 2 and 3).

**Table 2:** Statistical summary of all pairwise contrasts which tested the overall effect of each herbicide relative to the control for the three field experiments in 2015 and 2016.

Year	Experiment	Treatment	F	df	P-Value
2015	1	2,4-D	17.9	7,14	<0.001
		Command	23	7,14	<0.001
		Newpath	9.38	7,14	<0.001
		Ricebeaux	65.15	7,14	<0.001
2016	1	2,4-D	11.15	9,24	<0.001
		Bolero	12.64	9,24	<0.001
		Command	3.12	9,24	<0.001
		Newpath	8.19	9,24	<0.001
		Propanil	10.97	9,24	<0.001
		Ricebeaux	20.34	9,24	<0.001
2016	2	2,4-D	17.5	9,34	<0.001
		Bolero	14.25	9,34	<0.001
		Command	17.27	9,34	<0.001
		Newpath	15.75	9,34	<0.001
		Propanil	16.45	9,34	<0.001
		Ricebeaux	17.58	9,34	<0.001

Univariate analyses of the effects of herbicide treatment on the following response variables were performed: injury rating (Injury1 and Injury2), weed control rating (Weed1 and Weed2), insect densities (RWW, Whitehead, and Stinkbug) and Yield (Yield). Results showed significant injury (Objective 2) to rice in all herbicide treated plots in all three experiments at the first rating date seven days post application (Injury1); 2015 (F = 11.56, df = 4,20; P < 0.001), 2016(1) (F = 15.43; df = 6,24; P < 0.0001), 2016(2) (F = 29.58; df = 6,42; P < 0.0001) (Table 3). No injury was observed at the second rating (Injury2) in 2015 indicating plants in all treatments recovered from injury incurred by herbicide treatments by 21 days post application. Conversely, in the 2016 experiments the effect of herbicide treatment on level of injury was still significant at 21 days post application; 2016(1) (F = 12.08; df = 6,24; P < 0.0001), 2016(2) (F = 2.59; df = 6,42; P = 0.031). Plots treated with Ricebeaux and 2,4-D showed consistently higher injury relative to control plots over the three experiments. The injury level in Bolero treated plots was significantly higher than controls at the second injury rating 21 days post-application (Injury2) in the first experiment of 2016.

**Table 3:** Mean injury ratings (% injury ± S.E.) at two time points post-treatment (7 days post application and 21 days post application) for the field experiment in 2015 and the two field experiments in 2016 (2016(1) and 2016(2)).

Herbicide	2015 Injury1 7 DPA	2015 Injury2 21 DPA	2016 (1) Injury1 7 DPA	2016 (1) Injury2 21 DPA	2016 (2) Injury1 7 DPA	2016 (2) Injury2 21 DPA
Control	0 ± 0a	0 ± 0	0 ± 0a	0 ± 0a	0 ± 0a	0 ± 0a
2,4-D	10.00 ± 4.18a	0 ± 0	34.29 ± 2.97b	19.29 ± 1.30c	22.86 ± 2.86b	13.57 ± 3.57b
Bolero	N/A*	N/A*	12.87 ± 2.86a	14.29 ± 2.54bc	0 ± 0a	5.71 ± 2.97ab
Command	7.00 ± 2.55a	0 ± 0	2.14 ± 2.14a	2.14 ± 2.14a	1.43 ± 1.43a	4.29 ± 2.77ab
Newpath	1.00 ± 1.00a	0 ± 0	9.29 ± 3.52a	6.43 ± 3.22ab	1.43 ± 1.43a	5.00 ± 2.44ab
Propanil	N/A*	N/A*	12.86 ± 5.22a	2.14 ± 2.14a	1.43 ± 1.43a	7.86 ± 2.14ab
Ricebeaux	21.00 ± 2.45b	0 ± 0	27.86 ± 3.43b	16.43 ± 2.83c	1.43 ± 1.43a	5.00 ± 2.44ab

Values in the same column accompanied by different letters represent means that differed significantly with Tukey’s HSD test at α < 0.050. *N/A = Treatment was not applied in that year.

Univariate analysis revealed that herbicides significantly controlled weeds relative to controls at each rating date (Objective 3). In 2015 percent weed control at both rating dates was significantly higher in all herbicide treatments when compared to controls. Plots treated with Newpath and Ricebeaux showed significantly higher control than plots treated with 2,4-D or Command at both rating dates (Weed1 F = 188.5; df = 4,20; P < 0.001) (Weed2 F = 48.11; df = 4,20; P < 0.001) (Table 4).

**Table 4:** Mean weed control (% weed control ± S.E.) at two time points post-treatment (7 days post application and 21 days post application) for the field experiment in 2015 and both experiments in 2016 (2016 (1) and 2016 (2)).

Herbicide	2015 Weed1 7 DPA	2015 Weed2 21 DPA	2016 (1) Weed1 7 DPA	2016 (1) Weed2 21 DPA	2016 (2) Weed1 7 DPA	2016 (2) Weed2 21 DPA
Control	0 ± 0a	0 ± 0a	0 ± 0a	0 ± 0a	0 ± 0a	0 ± 0a
2,4-D	36.00 ± 1.00b	61.00 ± 7.31b	32.86 ± 12.86ab	8.57 ± 8.57ab	47.14 ± 11.49b	38.57 ± 10.33b
Bolero	N/A*	N/A*	54.29 ± 9.97bc	62.86 ± 5.10 cd	75.71 ± 3.69c	81.43 ± 7.54c
Command	31.00 ± 4.30b	48.00 ± 6.25b	15.71 ± 8.69ab	37.86 ± 15.39bc	57.41 ± 5.65bc	49.29 ± 9.16b
Newpath	84.00 ± 1.87c	84.00 ± 3.67c	54.29 ± 9.97bc	75.00 ± 8.17d	75.71 ± 2.97c	89.29 ± 4.93c
Propanil	N/A*	N/A*	48.57 ± 11.00bc	85.00 ± 3.93d	77.14 ± 3.60c	90.71 ± 2.02c
Ricebeaux	91.00 ± 4.00c	85.00 ± 4.47c	75.71 ± 2.97c	80.86 ± 4.74d	80.00 ± 3.09c	87.86 ± 3.76c

Values accompanied by different letters represent means that differed significantly with Tukey’s HSD test at α < 0.05. *N/A = Treatment was not applied in that year.

In the first experiment of 2016, percent weed control (Objective 3) in plots treated with Bolero, Newpath, Propanil, and Ricebeaux was significantly higher than in control plots at both rating dates. At the second rating date, Command-treated plots showed higher percent weed control than untreated plots. There were also significant differences among herbicide treatments, with Ricebeaux, Newpath, and Propanil consistently providing higher weed control than other treatments. 2,4-D did not control weeds when compared to untreated plots in this experiment at the first or second rating (Weed1 F = 8.19; df = 6,42; P < 0.0001) (Weed2 F = 19.40; df = 6,42; P < 0.0001) (Table 4).

In the second experiment of 2016, the 2,4-D treatment resulted in significantly higher weed control (Objective 3) than control plots, but weed control in 2,4-D treated plots was significantly lower than in all other herbicide-treated plots. At 21 days post-application, 2,4-D and Command provided significantly higher weed control relative to the control group, but significantly lower control than Bolero, Newpath, Propanil, and Ricebeaux (Weed1 F = 27.67; df = 6,42; P < 0.0001) (Weed2 F = 28.84; df = 6,42; P < 0.0001) (Table 4).

**Figure 5:**
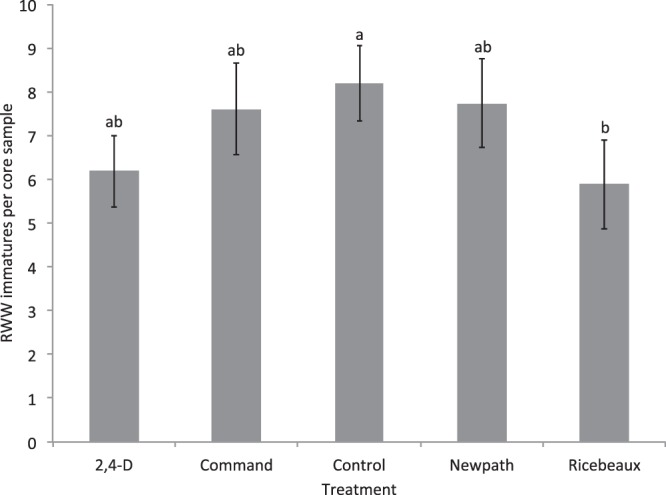
Mean densities of RWW immatures (numbers of larvae and pupae per core sample ± S.E.) in control and herbicide-treated plots over the four core sampling dates in 2015. Bars accompanied by different letters represent means that differed significantly by Tukey’s HSD test at α < 0.05.

Univariate analysis of data from core samples showed that densities of RWW (Objectives 2 and 3) in herbicide-treated plots differed significantly from controls in 2015 but not in 2016, though there were differences among treatments in 2016. In 2015, RWW densities were significantly lower in Ricebeaux-treated plots (Herbicide F = 3.54; df = 4,76; P = 0.011), and RWW densities differed significantly among core sampling dates with the third date the highest and first date the lowest (Date F = 35.77; df = 3,76; P < 0.001). The interaction between herbicide treatment and core sampling date was not significant (Herbicide*Date F = 1.31; df = 12,76; P = 0.232) (Fig. 5).

**Figure 6:**
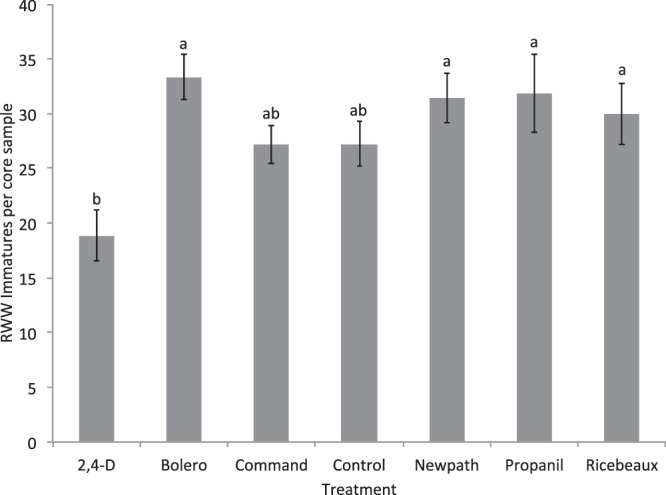
Mean densities of RWW immatures (numbers of RWW larvae and pupae per core sample ± S.E.) in control and herbicide-treated plots over the two core sampling dates in the first experiment of 2016. Bars accompanied by different letters represent means that differed significantly by Tukey’s HSD test at α < 0.05.

In the first experiment of 2016, there was a significant effect of herbicide on RWW densities, but none of the herbicide treatments differed from the control (Herbicide F = 3.88; df = 6,68; P = 0.002) Plots treated with 2,4-D showed the lowest RWW densities and densities in these plots were significantly lower than in Bolero-treated plots, which had the highest densities (Fig. 6). There was no significant effect of core sampling date or the interaction of herbicide and core sampling date (Date F = 0.09; df = 1,68; P = 0.770; Herbicide*Date F = 0.46; df = 6,68; P = 0.838).

In the second experiment of 2016, RWW densities differed significantly between the two core sampling dates, with densities higher on the second core sampling date (F = 6.88; df = 1,79; P = 0.011). Herbicide treatment and the interaction of herbicide treatment and core sampling date were not significant (Herbicide F = 0.65; df = 6,79; P = 0.693; Herbicde*Date F = 0.68; df = 6,79; P = 0.666).

Densities of whiteheads (Objectives 2 and 3) were measured in the two experiments in 2016, and differed significantly among herbicide treatments in both experiments, although no differences from controls were noted (2016 (1) F = 2.46; df = 6,42; P = 0.039) (2016 (2) F = 2.35; df = 6,42; P = 0.048). In the first experiment of 2016, plots treated with Ricebeaux (2.57 ± 0.43 whiteheads/plot) showed the lowest densities of whiteheads, and densities in these plots were significantly lower than in Command-treated plots (6.29 ± 1.23 whiteheads/plot), which had the highest densities. However, neither was significantly different from densities in control plots (3.71 ± 0.75 whiteheads/plot). In the second experiment of 2016 there were no significant differences in whitehead densities among treatments after Tukey adjustment.

Rice stink bug densities (Objectives 2 and 3) did not differ significantly among treatments in the first experiment in 2016 (F = 2.04; df = 6,42; P = 0.081), but were significantly affected by herbicide treatments in the second experiment (F = 2.94; df = 6,24; P = 0.018). More stink bugs were captured from plots treated with 2,4-D (8.43 ± 2.03 rice stink bugs/plot) and Bolero (8.29 ± 2.11 rice stink bugs/plot) than plots treated with Ricebeaux (3.89 ± 0.77 rice stink bugs/plot), although rice stink bug densities in all of these treatments did not differ from the control (5.57 ± 0.97 rice stink bugs/plot).

Finally, yield was significantly affected by herbicide treatments in the first experiment in 2016 (F = 2.69; df = 6,42; P = 0.027), but not in the second (F = 1.12; df = 6.42; P = 0.365). In the first experiment, results show that Bolero-treated plots (9.79 ± 0.25 kg/plot) had the lowest yield and Command-treated plots (12.49 ± 1.38 kg/plot) the highest. These treatments differed from each other, but not from the control (11.48 ± 0.16 kg/plot).

## Discussion

Applications of herbicides may affect herbivores in agroecosystems directly, by toxifying them, or indirectly, by inducing plant defenses or by altering the availability of non-crop plant hosts^13^. This study is one of only a few that has investigated direct and indirect effects of herbicides on herbivores simultaneously. Overall, the results from this series of experiments indicated that herbicides are capable of altering pest densities of both weeds and insects in rice, and the resulting changes in these communities may have impacts on yields.

The first objective of this study was to determine if applications of seven herbicides commonly used in rice in the southern U.S. caused mortality in RWW adults that were exposed by topical application or by feeding on leaves that had been recently treated with the herbicides. RWW adults are present and feeding in rice fields both before and after flooding of rice fields in the southern U.S, and are therefore present at the times that most herbicides are applied to rice fields in the southern U.S^20^. The herbicides caused significant mortality to RWW adults when applied topically at maximum label rates. This level of direct and sustained exposure is unlikely in the field as RWW adults are rarely found at the top of rice canopies, drop from plants when disturbed, and are capable of flying and swimming.

The feeding assays with herbicide-treated foliage likely better represent the type of exposure to which RWW adults are subjected in a field setting. RWW adults that were fed on rice with herbicide residue for the most part did not experience significant mortality. The exception to this was RWW adults fed on rice treated with Bolero. These insects experienced significant mortality when compared to RWW adults on non-treated rice tissue. The effect of consuming Bolero-treated rice tissue on RWW adults, however, did not translate to a reduction of immature RWW densities in Bolero-treated plots in the field experiments. In fact, Bolero-treated plots showed a trend toward higher densities of RWW immatures than the control plots. Thus, while herbicides, such as Bolero, may cause direct mortality of adults, this did not translate into reduced larval populations later in the season. This is likely due to the natural patterns of infestation of rice fields by RWW adults: relatively few adult weevils are present in fields before flooding, as they are still migrating from overwintering sites, and most arrive after flooding^20^.

The second objective was to determine if herbicide applications reduced pest densities by inducing resistance in the host plant. While evidence from this study does not provide substantial support for induction of defenses to all three insects by herbicides, it does provide support of low levels of resistance induced by herbicides to RWW. Of the herbivores studied, the RWW was most likely to be affected by herbicide-induced resistance, because it is the only insect of the three present in high numbers early in the growing season, when herbicides are applied^20^. RWW densities in herbicide-treated plots were significantly lower than control plots in one experiment (Ricebeaux-treated plots in 2015). However, there were instances in which densities of RWW in the various herbicide-treated plots differed significantly from each other, and this may have been related to the level of injury to the plants caused by herbicide treatments. Levels of induction of defenses in many plants are correlated with the amount of injury plants receive^31^. Over the three experiments in the current CCA suggested a negative correlation between injury and RWW density on five of eight core sampling dates. The herbicides 2,4-D, Bolero, and Ricebeaux consistently caused significant injury to rice plants. Ricebeaux-treated plots had lower RWW densities than many other treatments in most core samplings throughout the three experiments. In the first experiment of 2016 RWW densities in 2,4-D-treated plots were significantly lower than densities than several other herbicide-treated plots including Bolero and Ricebeaux. The injury caused by 2,4-D in this experiment was likely due to inappropriate timing of this herbicide, which is labeled for use before seeding or after the permanent flood.

The herbicides used in these experiments have different modes of action, and were used because mode of action could conceivably influence toxicity to insects, induction of plant defenses, and effectiveness of weed control. Bolero and Ricebeaux, two of the herbicides with the largest effect on RWW, both contain photosynthesis inhibitors, which reduce electron transport and have various effects on host plant quality. In rice, for example, pesticide-induced susceptibility to brown planthopper was correlated with reduced photosynthesis^30^. Thus, herbicide injury can affect plant physiology in ways that do not involve induction of resistance-related traits, and these changes in plant physiology may be relevant to herbivore performance.

Unlike RWW, stemborers and rice stink bugs are typically present in rice fields in high numbers later in the season and are less likely to be affected by herbicide-induced resistance, which, like other types of induced resistance, probably decays over time^32–34^. The results of the field experiments were consistent with a lack of herbicide-induced effects on these insects, as the only significant relationship between injury ratings and whitehead densities occurred in 2016 when injury was extremely low, and no significant relationship between injury and stink bug densities were found. Overall, our results suggest that the herbicides used in this study may have induced low levels of resistance to the RWW, but not to the other insect pests. It may be that high levels of induction were not achieved because overall injury from herbivores was low. The low levels of injury from herbicides was not surprising, since these herbicides were developed with the intent of causing little injury to the crop.

The third objective of this study was to determine if changes in plant (weed) populations caused by herbicide applications altered densities of subsequent insect pest populations. The herbivores involved in this study preferentially utilize grasses, including common weeds in rice. Densities of stemborers and rice stink bug were expected to be more affected by changes in weed densities, than by the other two routes of herbicide impact as these insects are not present in early season rice when herbicide applications are most frequently made. Rather, they attack rice in mid- to late-season, well after herbicide applications are made and when differences in weed densities are most pronounced.

Results provide modest support that alteration in weed densities affected densities of these pests. The CCA showed that in twelve of sixteen core sampling dates and rating combinations the relationship between weed control and RWW densities was negative; that is, RWW densities decreased as weed densities decreased. This was not further supported by simple linear regressions as only two of the sixteen combinations showed a significant negative relationship on the univariate level. In contrast, relationships among weed densities and densities of mid- to late-season pests were stronger. The CCA showed that overall densities of whiteheads and rice stink bugs were positively correlated with weed densities in six of eight insect samplings and weed rating combinations. While simple linear regressions showed that densities of whiteheads and rice stink bugs were not significantly correlated with weed densities in experiments performed in 2016, there was some support at the univariate level. Command-treated plots had the lowest weed control rating (aside from the control) at seven days post-application and the highest whitehead count, while Ricebeaux-treated plots had the highest weed control rating and the lowest whitehead count, suggesting that plots with higher weed densities had higher densities of borers. The relationship did not hold, however, for the second weed rating at 21 days post-application. In all three experiments, for five of six weed control rating dates, all herbicides were shown to be capable of significantly controlling weed pests. Therefore, overall weed densities in the herbicide-treated plots were low, and this may have prevented a robust investigation of the effect of weed density on rice stink bug and stemborer densities.

Differences in yields were observed among herbicide treatments. CCA of both experiments in 2016 showed a negative relationship between injury and yield. Results of simple linear regression in both experiments in 2016 support this result and showed a significant negative relationship between level of injury to rice and yield. This suggests that herbicide injury may have contributed to yield reduction. In the second experiment of 2016, injury was greatest in plots treated with 2,4-D, from 13–22%, and yields from 2,4-D-treated plots were the lowest of all groups. The first experiment of 2016 also showed that Bolero-treated plots had significantly lower yields than Command-treated plots. This may have been attributable to the fact that Bolero-treated rice received as much as 85% more injury than Command-treated plots in this experiment. It may also be explained by the high rates applied and inappropriate timings of applications.

In the second experiment of 2016, CCA showed a positive correlation between weed control and yield for three of four rating dates. The simple linear regressions supported this result and showed a significant increase in yield with increasing weed control. This may have been due to reductions in plant competition, but may also be attributed to lower insect pest densities in plots with lower weed densities. The combination of herbicide injury and insect herbivory has previously been shown to have negative effects on plant health. For example, weed management was shown to be more effective when volunteer potatoes, considered to be weeds, were defoliated by Colorado potato beetles, *Leptinotarsa decemlineata* Say, after herbicide-stress, relative to control of weeds by beetles on plants that were not herbicide-stressed^35^.

While there have been many studies investigating the effects of herbicides on insect communities, the vast majority focused on direct effects on insect mortality or fecundity and indirect effects due to changes in weed populations^36^. The literature is not, however, devoid of investigations of herbicide-induced resistance to herbivores. One study suggested 2,4-D induced susceptibility in corn, *Zea mays*, to two insects and one pathogen^37^. Additionally, studies investigating herbicide-induced stress in the plant and subsequent effects of herbivory have been conducted. Phosphonate herbicides have been shown to have a dual effect as both herbicides and insecticides to the black bean aphid, *Aphis fabae* Scopoli (Hemiptera: Aphididae), when applied systemically in broad bean, *Vicia faba*^38^. There are few if any other studies that have combined investigations of all three routes by which herbicides may alter insect communities. There is a need for additional studies investigating direct and indirect effects simultaneously in order to make clearer inferences.

The use of herbicides is ubiquitous in modern agroecosystems, and thus it is important to understand effects of herbicide applications on arthropod communities in addition to their effects on the weeds they are intended to control. This study is one of the few studies to simultaneously investigate multiple routes by which herbicides could potentially affect arthropods and crop yields. Use of herbicides had minor and inconsistent impacts on insect pest densities in rice, whereas rice yields were negatively impacted by herbicide injury and presence of weeds. Although further studies in other crops are needed to investigate the relative importance of the various routes by which herbicides can impact populations of herbivores and crop yields, this study overall reinforces the importance of appropriate weed management in agricultural systems, as increases in weed and insect densities can negatively affect yield, as can crop injury resulting from improper use of herbicides.

## Materials and Methods

### Study site and rice culture

All experiments were conducted at the Louisiana State University Agricultural Center H. Rouse Caffey Rice Research Station (30.231422°N and −92.379583°W, 7 m asl). The soil type at this site is Crowley silt loam with a pH of 7.1 and 12% organic matter. Fields at this site have been in a rice-fallow rotation for over 30 years. Rice in all experiments was drill-seeded in plots measuring 1.3 m by 5.5 m with 7 rows of rice at 17.8 cm spacing. Seeding rate was 67 kg/ha. The rice variety ‘CL111’ was used in all experiments. ‘CL111’ is an imidazolinone-tolerant long-grain inbred variety developed by the rice breeding program at the H. Rouse Caffey Rice Research Station^39^. Plots were fertilized with 134 kg/ha N applied pre-flood and 67 kg/ha P and K applied in the previous fall. Fertilization and disease control practices followed recommendations for drill-seeded rice in southwest Louisiana^40^. Dates of all planting, fertilization, and flooding activities can be found in Supplemental Table 1. Plots were harvested at grain maturity with a mechanical combine and yield was adjusted to 12% moisture.

### Rice water weevil mortality experiments (objective 1)

To investigate direct effects of herbicides on survival of adult RWW, adults were exposed either by direct topical application or by exposure to leaves treated with herbicides. RWW adults were collected from untreated border plots and used in experiments the same day as collection. For both experiments all herbicides were applied at their highest label rate. Herbicide treatments used in the 2015 experiment included ‘Weed Rhap A-4D’ (2,4-D), ‘Command 3ME’ (Command), ‘Newpath’ (Newpath), ‘Ricebeaux’ (Ricebeaux), ‘Propanil 4SC’ (Propanil), ‘Bolero 8EC’ (Bolero) and a control (Control), which was sprayed with water only. Information concerning the trade names, manufacturers, active ingredients, modes of action, rates, and typical timings for applications of each of these herbicides is provided in Supplemental Table 1. To test the effects of topical exposure, four petri dishes for each herbicide, each containing ten field-collected RWW adults, were placed on the ground and lids removed. Herbicides were applied using a backpack sprayer pressurized with CO_2_ and calibrated to deliver 140 L/ha at 207 kPa through three 1002 flat fan nozzles TeeJet® at 51 cm spacing. Mortality was assessed 24 hours after spraying. To assess mortality, weevils in each petri dish were observed for 15–30 minutes. RWW adults that did not display coordinated movement within that time were removed from the dish, and recorded as dead^41^.

To test the effects of exposure of RWW adults to leaves treated with herbicide, weevils were fed on herbicide-treated leaf material immediately after treating plants with herbicides. This was done by spraying one field plot by backpack sprayer with each herbicide at the same rate as described above. Rice foliage from these plots was immediately cut from the plants with scissors, and placed in a petri dish lined with moistened filter paper for adult feeding. Approximately 20 leaves were placed in each dish to ensure that weevils would not be food-limited. Ten RWW adults were then added to each of four dishes for each herbicide. The petri dishes were then moved to a colony rearing room with 16:8 L:D, 22 °C, 70% RH until mortality was assessed. Mortality was assessed one day after herbicide applications described above.

### Herbicide treatments for field experiments (objective 2 and 3)

Herbicide treatments used in the 2015 experiment included ‘Weed Rhap A-4D’ (2,4-D), ‘Command 3ME’ (Command), ‘Newpath’ (Newpath), ‘Ricebeaux’ (Ricebeaux), and an unsprayed control (Control). A randomized complete block design with five blocks and one replicate of each treatment within each block was used. Herbicides were applied one day prior to flooding, when plants had reached the four-leaf stage. Although this timing of application differs from standard timing for herbicide applications in rice, which generally include a pre-emergence application followed by one or more post-flood applications at or near the time of flooding, this timing was used to standardize treatment applications and facilitate comparison of effects on insects. All herbicides were applied at their highest label rate. Information concerning the trade names, manufacturers, active ingredients, mode of action, rates, and typical timings for applications of each of these herbicides is provided in Supplemental Table 1. Herbicides were applied using a backpack sprayer pressurized with CO_2_ and calibrated to deliver 140 L/ha at 207 kPa through three 1002 flat fan nozzles TeeJet® at 51 cm spacing.

Treatments in 2016 included all herbicides used in 2015. In addition to these herbicides, the herbicides ‘Propanil 4SC’ (Propanil) and ‘Bolero 8EC’ (Bolero) were also used. The two field experiments conducted in 2016 followed the same experimental design as the 2015 experiment, with the exception of the addition of the Propanil and Bolero treatments and two blocks, for a total of seven blocks in each experiment, and one replicate of each treatment in each block. The active ingredients of Propanil and Bolero are both present in Ricebeaux. Therefore, in order to determine if either alone was responsible for the effects of Ricebeaux observed in 2015, both were used in 2016 in addition to Ricebeaux. Information regarding dates of application and flooding can be found in Supplemental Table 2.

### Insect sampling (objective 2 and 3)

All experiments relied on natural infestations of the insects under study. Insect densities were not measured prior to herbicide applications. Pre-treatment assessments of RWW populations were not made because infestations of larvae occur only after rice is flooded, and pre- and post-flood densities of RWW adults are not associated with post-flooding larval densities^20,42^. Pre-treatment densities of stemborers and rice stink bugs were not assessed because these insects do not infest rice fields until much later in the growing season (see description of pest life cycles above).

The procedure for determining densities of RWW larvae and pupae on roots after flooding involves removing soil/root core samples from plots and counting larvae and pupae associated with roots and has been described in detail elsewhere^43^. Larval densities are expressed as the numbers of immatures per core sample. Stemborer and rice stink bug densities were measured in the 2016 experiments only. Densities of whiteheads found in plots shortly after heading were used to estimate stemborer infestation levels. Whitehead counts were made by visual inspections within one week of anthesis, approximately two months after herbicide applications. Populations were a mixture of the three stemborer species mentioned above, but larvae were not identified to species. The procedure used for estimating rice stink bug density consisted of sweeping plots with 38 cm sweep nets. The length of each plot was swept with ten consecutive sweeps across the plot and numbers of adult and immature bugs captured in nets were counted. The total number of rice stink bugs per plot was used for analysis. Sweeps were made within one week of anthesis, approximately two months after herbicide application.

### Rating of rice injury and weed control (objective 2 and 3)

Visual assessments of rice injury and weed control resulting from herbicide treatments were made one and three weeks after application of herbicide. Ratings for injury were based on chlorosis and necrosis of foliage and plant height on a scale of 0 to 100, with 0 = 0 injury and 100 = complete plant death. The scale for weed control was also 0 to 100, with control plots representing the standard for zero weed control.

### Statistical analyses

All analyses were performed in SAS version 9.4 (SAS Institute 2013). Means, standard deviations, and standard errors were determined in PROC MEANS. Poisson distribution and log link were used for all count data. All continuous data were tested for normality with PROC UNIVARIATE and the Shapiro-Wilk test (P < 0.05).

For the two RWW mortality experiments (Objective 1), each petri dish containing ten weevils was considered a replicate and the percentage of dead weevils in each dish was calculated and used for analyses. Mortality data were analyzed by generalized linear mixed models using PROC GLIMMIX, with herbicide treatment as the independent variable. Means were tested for significant differences using Tukey’s HSD (P < 0.05). This analysis tested the hypothesis that applications of herbicides directly reduced RWW survival.

Each of the three field experiments were analyzed separately. The models described below tested the hypotheses that herbicide-induced resistance indirectly altered RWW densities, borer densities, rice stink bug densities, and yields (Objective 2) or that herbicide-induced changes in in weed communities altered densities of these insects and yields (Objective 3), but the analyses do not allow these two hypotheses to be distinguished.

For the 2015 experiment, multivariate approaches were utilized followed by appropriate univariate tests. Response variables included RWW densities in each of the four core samplings (RWW1, RWW2, RWW3, and RWW4), two ratings of weed density (Weed1 and Weed2), and two ratings of rice injury (Injury1 and Injury2). Each core sampling was maintained as a separate response variable to allow for analysis of effects of time. Canonical correlation analysis (CCA)^44,45^ in PROC CANCOR was first performed to determine if significant relationships existed between response variables. Biological relationships were inferred from the canonical cross loadings for individual variables from the two sets of variates. To analyze the twov2016 experiments, CCA was conducted as for the 2015 experiment, but with the addition of three response variables, whitehead densities (Whitehead), rice stink bug densities (Stinkbug), and plot yields (Yield). CCA was performed with one group including response variables RWW1, RWW2, Whitehead, Stinkbug, and Yield, and the second group including response variables Injury1, Injury2, Weed1, and Weed2. One outlier was removed from the first experiment in this year. While CCA is a very informative and appropriate analysis for this experimental design, biological effects are difficult to portray visually. Due to this weakness, and in order to provide additional support for the results of the CCA, simple linear regressions were performed using PROC REG for both 2015 and 2016 experiments to reveal the nature of direct relationships between variables of interest. These regression analyses were performed under circumstances in which neither variable was measured without error because they were both response variables of the herbicide treatments. The CCA and simple linear regression analyses test potential effects of both induced resistance and of altered weed community. In each analysis (CCA and simple linear regression), the level of injury to rice and level of weed control were compared to the subsequent densities of RWW. As CCA and simple linear regression analyses utilize only the response variables in the dataset there is no analysis of effects of herbicide treatments involved in either. One outlier was removed from the CCA in 2015. Simple linear regression was performed as described above for both experiments in 2016 even though the canonical correlation analysis was only marginally significant in the second experiment in this year.

Additional multivariate analyses consisting of a MANOVA followed by pairwise contrasts of each herbicide treatment to the control were performed separately for each experiment in PROC GLM (Objectives 2 and 3). Block was used as a random effect and herbicide as a fixed effect in these analyses. Results of MANOVA and pairwise contrasts were considered significant when the p-value from Wilk’s Lambda test was less than 0.05. Significant results in the pairwise contrasts indicated that one or more response variables showed significant differences from the control.

Response variables significant at the multivariate level were analyzed by univariate generalized linear mixed models in PROC GLIMMIX. Means, standard deviations, and standard errors were determined in PROC MEANS. The Poisson distribution and log link were used for all count data (insect densities). RWW densities were determined on four separate core sampling dates for 2015 and two separate core sampling dates in 2016. In order to analyze these data, block was used as a random effect and core date, herbicide and the interaction of core date and herbicide were used as fixed effects. For densities of whiteheads and rice stink bug, block was used as a random effect and herbicide as a fixed effect. The identity link and Gaussian distribution were used for continuous data (yields), and block was used as a random effect and herbicide as fixed effect.

## Supplementary information

Supplementary Tables

## Data Availability

The datasets generated and/or evaluated during the current study are available from the corresponding author on request.
