# Supplementary material for: Direct and Indirect Effects of Herbicides on Insect Herbivores in Rice, Oryza sativa
Source: Sci Rep. 2019 May 6;9:6998. doi: 10.1038/s41598-019-43361-w (PMC6502833; doi:10.1038/s41598-019-43361-w)
Supplement: Supplementary file 1 — Supplementary Tables [file 41598_2019_43361_MOESM1_ESM.docx]

Direct and Indirect Effects of Herbicides on Insect Herbivores in Rice, Oryza sativa

Emily C. Kraus^1,2^ and Michael J. Stout^1^

^1^ Louisiana State University AgCenter Baton Rouge Louisiana

^2^ Corresponding author, email: krausec07@gmail.com

Supplemental Table 1

| Trade Name | Active Ingredient | Mode of Action | Rate Range | Rate Used | Surfactant Y/N | When to Apply |
| --- | --- | --- | --- | --- | --- | --- |
| Weed Rhap A-4D | Dimethylamine salt of 2,4-dichloro-phenoxyacetic acid | Growth Regulator | 1-2.5 pints/acre | 2.5 pints/acre | Y | 4 weeks prior to planting or late tillering stage |
| Command 3ME | Clomazone | Pigment Inhibitor | .4-.6lbs/acre | .8lbs/acre | Y | Prior to planting to 7 days after planting |
| Bolero 8EC | Thiobencarb | Photosynthesis Inhibitor | 2-4 pts/acre | 4 pts/acre | N | Prior to planting not past 2-3 leaf stage on wet soil only |
| Newpath | Ammonium salt of imazethapyr | ALS Inhibitor | 4-6 floz/acre | 6 floz/acre | Y | 2-5 leaf stage; Requires two applications for control |
| Propanil 4SC | 3’, 4’-Dichloropropionanilide | Lipid Synthesis Inhibitor | 3-6qts/qcre | 6qts/acre | N | 1-4 leaf stage |
| Ricebeaux | Propanil and Thiobencarb | Lipid Synthesis Inhibitor and Photosynthesis Inhibitor | 3-5.3 qts/acre | 5.3qts/acre | N | 1-6 leaf stage |
| Low Foam Surfactant | Alkyl Polyoxyalkylene Ether | N/A | N/A | N/A | N/A | N/A |

Supplemental Table 2

| Year | Experiment | Planting Date | Fertilization Date | Application Date | Flooding Date | Rating 1 Date | Rating 2 Date | Core 1 Date | Core 2 Date | Core 3  Date | Core 4 Date | WH Count Date | RSB Sweep Date | Harvest Date |
| --- | --- | --- | --- | --- | --- | --- | --- | --- | --- | --- | --- | --- | --- | --- |
| 2015 | 1 | 4-May | 9-Jun | 8-Jun | 10-Jun | 15-Jun | 30-Jun | 25-Jun | 29-Jun | 7-Jul | 15-Jul | x | x | x |
| 2016 | 2 | 29-Mar | 11-May | 11-May | 12-May | 18-May | 2-Jun | 1-Jun | 8-Jun | x | x | 5-Jul | 5-Jul | 11-Aug |
| 2016 | 3 | 9-May | 2-Jun | 2-Jun | 3-Jun | 17-Jun | 5-Jul | 20-Jun | 28-Jun | x | x | 27-Jul | 27-Jul | 24-Aug |
